# *Drosophila* E-Cadherin Functions in Hematopoietic Progenitors to Maintain Multipotency and Block Differentiation

**DOI:** 10.1371/journal.pone.0074684

**Published:** 2013-09-05

**Authors:** Hongjuan Gao, Xiaorong Wu, Nancy Fossett

**Affiliations:** Center for Vascular and Inflammatory Diseases and the Department of Pathology, University of Maryland School of Medicine, Baltimore, Maryland, United States of America; University of Frankfurt - University Hospital Frankfurt, Germany

## Abstract

A fundamental question in stem cell biology concerns the regulatory strategies that control the choice between multipotency and differentiation. *Drosophila* blood progenitors or prohemocytes exhibit key stem cell characteristics, including multipotency, quiescence, and niche dependence. As a result, studies of *Drosophila* hematopoiesis have provided important insights into the molecular mechanisms that control these processes. Here, we show that E-cadherin is an important regulator of prohemocyte fate choice, maintaining prohemocyte multipotency and blocking differentiation. These functions are reminiscent of the role of E-cadherin in mammalian embryonic stem cells. We also show that mis-expression of E-cadherin in differentiating hemocytes disrupts the boundary between these cells and undifferentiated prohemocytes. Additionally, upregulation of E-cadherin in differentiating hemocytes increases the number of intermediate cell types expressing the prohemocyte marker, Patched. Furthermore, our studies indicate that the *Drosophila* GATA transcriptional co-factor, U-shaped, is required for E-cadherin expression. Consequently, E-cadherin is a downstream target of U-shaped in the maintenance of prohemocyte multipotency. In contrast, we showed that forced expression of the U-shaped GATA-binding partner, Serpent, repressed E-cadherin expression and promoted lamellocyte differentiation. Thus, U-shaped may maintain E-cadherin expression by blocking the inhibitory activity of Serpent. Collectively, these observations suggest that GATA:FOG complex formation regulates E-cadherin levels and, thereby, the choice between multipotency and differentiation. The work presented in this report further defines the molecular basis of prohemocyte cell fate choice, which will provide important insights into the mechanisms that govern stem cell biology.

## Introduction

Stem cells have the dual capacity to self-renew and differentiate, thereby replenishing the stem cell pool and producing the entire spectrum of cells that form a given tissue. These characteristics allow stem cells to maintain tissue homeostasis throughout the life of an organism by mounting a response against environmental assaults and replacing lost or damaged tissue [1]–[3]. A central problem in stem cell biology involves identifying the regulatory strategies that determine how stem cells retain multilineage developmental potential (multipotency) or enter the differentiation pathway. The *Drosophila* hematopoietic system has become an important model for identifying molecular mechanisms that control the choice between multipotency and differentiation [4]–[6].

*Drosophila* hematopoietic progenitors or prohemocytes share key characteristics with mammalian hematopoietic stem cells, including quiescence, multipotency, and niche-dependence [5]–[8]. Prohemocytes give rise to all three *Drosophila* blood lineages [9]. These include the following: 1) plasmatocytes are operational macrophages that mediate phagocytosis of bacterial pathogens and apoptotic bodies; 2) crystal cells are named for their crystalline inclusion bodies, and are involved in wound healing; and 3) lamellocytes are normally rare blood cells that are produced in large numbers in response to various types of immune challenge [10]–[12].

*Drosophila* hematopoiesis takes place during two spatially and temporally distinct periods or waves, which is similar to the pattern seen in vertebrate blood systems [13], [14]. The first wave begins in the embryonic head mesoderm and continues within the larval hemocoel [14]. The second wave takes place during the larval stages in a specialized organ known as the lymph gland [9], [14]. The lymph gland is a bi-lateral organ that includes one pair of primary lobes and a series of secondary lobes flanking the larval heart [4]. The primary lobe is organized into three regions or zones with distinct hematopoietic functions. The first is the Posterior Signaling Center (PSC), which functions as a niche to maintain prohemocyte quiescence and multipotency. The second or medullary zone contains the prohemocyte population. The third region, or cortical zone, contains all three differentiated blood cell types. Additionally, cortical zone hemocytes signal to the medullary zone to maintain prohemocyte multipotency [4]–[6], [15], [16]. These findings illustrate the importance of this zonal arrangement in identifying the origin of signal transduction pathways and transcriptional regulators that direct the choice between multipotency and differentiation.

E-cadherin is the founding member of a large evolutionarily conserved family of calcium-dependent transmembrane proteins that are the principal components of adherens junctions [17], [18]. E-cadherin-mediated intercellular adhesion is required for embryonic development and for maintaining tissue integrity throughout the life of the organism [17]–[20]. Recent studies using mammalian model systems have identified E-cadherin-mediated stem cell-stem cell adhesion as a critical regulator of potency [21]–[26]. Embryonic stem cells (ESCs) and induced pluripotent stem cells (iPSCs) have the potential to produce all tissue types and are said to be pluripotent. E-cadherin is required for ESC pluripotency, self-renewal, and survival; whereas, loss of E-cadherin promotes differentiation [22], [24], [27]. Furthermore, gene expression profiles of ESCs indicate that E-cadherin plays a central role in regulatory pathways that are associated with a wide range of cellular processes involved in stem cell homeostasis [25]. E-cadherin has also been shown to be a fundamental component of the regulatory network that can generate iPSCs from terminally differentiated murine fibroblasts [21], [23], [28]. Additionally, studies using mammalian model systems suggest that E-cadherin promotes pluripotency by connecting extrinsic signals to the intrinsic stem cell transcriptional network [24]–[26]. Thus, E-cadherin functions as a key regulator of multipotency. This is an expanded role for E-cadherin beyond that observed in *Drosophila* germline stem cells and mammalian hematopoietic stem cells. In these systems, cadherins have been shown to function primarily as anchors that tether stem cells to the niche [29]–[33].

E-cadherin is uniformly expressed on the surface of *Drosophila* prohemocytes in a pattern similar to mammalian ESCs and iPSCs [4], [23], [34], [35]. The tight compaction of prohemocytes within the medullary zone is most likely the result of E-cadherin-mediated cell adhesion. Support for this interpretation comes from data that shows compaction is lost when E-cadherin is downregulated during prohemocyte differentiation [4], [16], [34]. However, the cellular role of E-cadherin in prohemocytes has not been previously reported. In this report, we show that E-cadherin is required to maintain prohemocyte multipotency and block differentiation of all three *Drosophila* blood cell types. Furthermore, mis-expression of E-cadherin in differentiating hemocytes upregulated the prohemocyte marker, Patched (Ptc), and disrupted the boundary between the cortical and medullary zones. These two E-cadherin-mediated processes in the fly are similar to those seen in mammalian pluripotent stem cells. Previous work from our laboratory showed that the Friend of GATA (FOG) transcriptional co-regulator, U-shaped (Ush), also maintains multipotency and blocks differentiation of all three blood cell types [34]. Based on these similarities, we investigated the potential regulatory links between Ush and E-cadherin. Our results indicate that E-cadherin acts downstream of Ush to maintain prohemocyte multipotency and block differentiation. Moreover, forced expression of the Ush GATA-binding partner, Serpent (Srp), repressed E-cadherin expression and promoted lamellocyte differentiation. This observation indicates that Ush maintains E-cadherin expression by blocking the inhibitory activity of Srp. Collectively, these findings suggest that control of GATA:FOG complex formation regulates E-cadherin levels and, thereby, the choice between multipotency and differentiation.

The work presented in this report further characterizes an important model system, which can be used to expand our understanding of how E-cadherin mediated stem cell-stem cell adhesion promotes potency *in vivo*. This is of particular importance given that E-cadherin apparently links extrinsic signals to the ESC core pluripotency machinery. Additionally, there is a growing body of evidence that GATA factors can influence E-cadherin function and, thereby, control epithelial to mesenchymal transition during normal development and metastatic cancer [36]–[39]. In this report, we provide evidence that the interaction between GATA and FOG may be a key regulator of this important adhesion molecule.

## Materials and Methods

### Fly Strains

*w^1118^* or *y w^67c23^* flies served as the wild-type stock for these studies. The following strains were generous gifts from colleagues: *UAS-E-cadherin* from G. Longmore (Washington University); *Tep4-Gal4* from T. Tokusumi and R. A. Schulz (University of Notre Dame); *domeless-Gal4* from M. Crozatier (University Paul Sabatier); *hemolectin-Gal4* from S. A. Sinenko (University of California, Los Angeles); *y^1^ w; ush^vx22^/CyO, y^+^* and *y^1^ w; ush^r24^/CyO, y^+^* from R. P. Sorrentino and R. A. Schulz (Saint Leo University and University of Notre Dame, respectively). *UAS-E-cadhern^RNAi^* transformant number 27082 and *UAS-U-shaped^RNAi^* transformant number 104102 were obtained from the Vienna *Drosophila* RNAi Center (VDRC). The following strains were obtained from the Bloomington Stock Center: *y^1^ v^1^;UAS-E-cadherin^RNAi^/TM3, Sb^1^*, *cn^1^ shg^2^ bw^1^ sp^1^/CyO*, *odd^01863^ cn^1^/CyO ry^506^*. The following strains have been described previously: *y^1^ w; odd^01863^/CyO, y^+^*; *UAS-serpentNC*, *y^+^*; *UAS-serpentNC^V421G^* [40], [41]. *w*; *UAS-U-shaped^RNAi^*; *UAS-E-cadherin* males were produced using the following strategy: First, *w*; *UAS-U-shaped^RNAi^*; TM3, Sb/TM6, Tb and *w*; *Sco/SM6, Roi*; *UAS-E-cadherin* stocks were generated and mated. Second, male and virgin female progeny with the *w*; *UAS-U-shaped^RNAi^/SM6, Roi*; *UAS-E-cadherin/*TM3, Sb genotype were selected and mated. Third, male progeny with the *w*; *UAS-U-shaped^RNAi^*; *UAS-E-cadherin* genotype were selected and used in these studies.

### Gene Expression Analyses

Gene expression analyses were conducted using lymph glands from mid-third instar larvae (collected 92 to 100 hours after egg laying). However, as indicated in specific experiments, gene expression analyses were also conducted using either early-third instar larvae (collected 74 to 82 hours after egg laying) or late-third instar larvae (collected 112 to 120 hours after egg laying). All control and experimental samples were age matched and cultured on standard media at 23°C. The UAS/Gal4 binary system [42] was used to express transgenes in a tissue-specific manner. Controls for each experiment included the Gal4 drivers crossed to *w^1118^* or *y w^67c23^* mates. In general, we used *domeless-Gal4* to drive *UAS-transgene* expression in prohemocytes. However, *domeless-Gal4* could not be used to drive *UAS-SrpNC* transgenes because this combination was lethal prior to the third larval instar. In this case, we used *Tep4-Gal4* in place of *domeless-Gal4*. Finally, the *Tep4-Gal4* and *domeless-Gal4* drivers were used interchangeably with the *UAS-E-cadherin^RNAi^* transgene because both driver/responder pairs showed the same capacity to reduce the prohemocyte pool and increase lamellocyte differentiation. Trans-heterozygous larvae were generated by crossing *cn^1^ shg^2^ bw^1^ sp^1^/CyO* or *cn^1^ shg^E17B^ bw^1^ sp^1^/CyO* to *y^1^ w; ush^vx22^/CyO, y^+^* to produce *y^1^ w*/Y*; shg/CyO, y^+^* males. These males were then crossed to *y^1^ w; ush^vx22^/CyO, y^+^* or *y^1^ w; odd^01863^/CyO, y^+^* virgin females. *y^1^ w; shg/odd* or *y^1^ w; shg/ush* trans-heterozygotes were obtained by selecting larvae with yellow mouth hooks as previously described [34]. Additionally, standard methods were used to recombine either the *ush^vx22^* or the *shg^2^* allele with wild-type chromosomes. These recombined chromosomes were then retested as heterozygotes and trans-heterozygotes for aberrant lamellocyte differentiation.

### Immunofluorescence

The dissection and fixation of larval lymph glands were performed as previously described [34]. Rabbit anti-Odd-skipped (Odd) was a generous gift from J. Skeath (Washington University School of Medicine, [43]) and used at a 1∶4,000 dilution. The following mouse antibodies directed against specific hemocyte antigens were generous gifts from I. Ando (Biological Research Center of the Hungarian Academy of Sciences) and used at the indicated dilutions: P1 (Nimrod; [44]), 1∶50 and L1 (Attilla; [45]), 1∶50. Rabbit anti-prophenoloxidase A1 (anti-ProPO) was a generous gift from F. C. Kafatos (EMBL, [46]) and used at a 1∶100 dilution. Mouse anti-Peroxidasin was a generous gift from L. Fessler and J. Fessler (UCLA, [47]) and was used at 1∶1,000 dilution. Rabbit anti-U-shaped was used at a 1∶4,000 dilution [48]. Mouse anti-Patched and rat anti-DE-cadherin antibodies were obtained from the Developmental Studies Hybridoma Bank and used at a concentration of 10 µg/ml. Rabbit anti-GFP (Invitrogen) was used at a 1∶10,000 dilution. Alexafluor-555- or −488-conjugated secondary antibodies directed against rabbit, mouse, or rat (Invitrogen) were used at a 1∶2,000 dilution. Fluorescence was captured, analyzed, and recorded using Olympus confocal microscopy or Zeiss Axioplan optics. The relative expression of medullary zone markers was assessed by determining the area of labeled cells and dividing by the total primary lobe area for several cross sections. Plasmatocyte and crystal cell counts were also divided by the total primary lobe area to normalize for differences in lymph gland size. Blood cell counts were analyzed using Zeiss Axioplan software as previously described [15], [41]. Relative Ush expression levels were determined from the densitometric mean values calculated for fluorescent antibody staining using Zeiss Axiovision software as previously described [34]. The statistical significance was evaluated using a two-tailed Student’s t-test. In our hands, control lymph glands have an average of 1 lamellocyte per lymph gland lobe. However, lamellocytes can form large aggregates making it difficult to obtain accurate cell counts. For this reason, we scored primary lymph gland lobes positive for aberrant lamellocyte differentiation when aggregates were greater than 300 µm^2^ or more than 5 individual lamellocytes were visible [34]. Statistical significance was then evaluated using aberrant differentiation as a categorical variable for experimental and control samples in 2×2 contingency tables. P values were calculated using the two-tailed Fisher’s exact test. At least 24 primary lymph gland lobes were sampled for each assay, consisting of 12 control and 12 experimental samples.

## Results

### E-cadherin is Required to Maintain the Medullary Zone Prohemocyte Population

**Figure 1:**
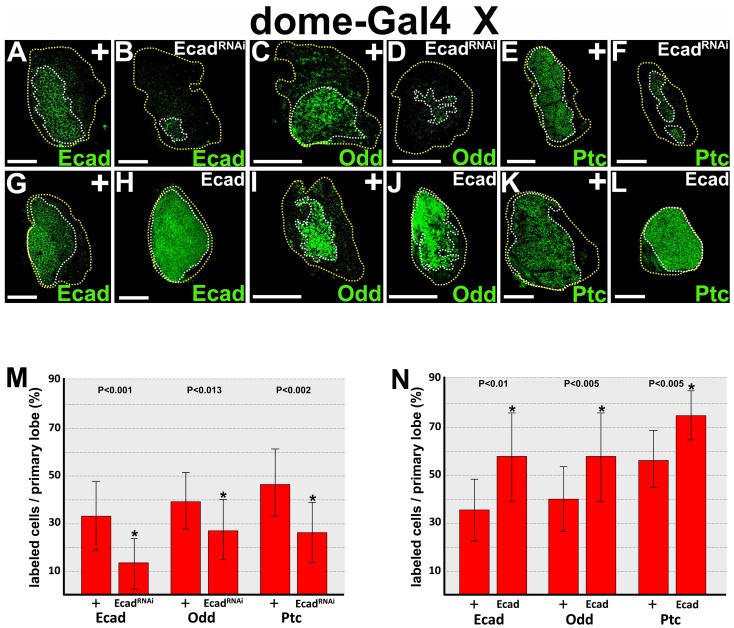
E-cadherin maintains the prohemocyte pool. (**A–F**) Loss of E-cadherin (Ecad) function reduces the prohemocyte population. *dome-Gal4* females were crossed to (**A,C,E**) control (+) or (**B,D,F**) *UAS-Ecad^RNAi^* (Ecad^RNAi^) males to knockdown Ecad expression in prohemocytes. (**A,B**) *dome-Gal4* driven Ecad^RNAi^ dramatically reduced the number of Ecad-expressing cells. (**C–F**) Targeted knockdown of Ecad reduced expression of the prohemocyte markers (**D**) Odd and (**F**) Ptc compared to (**C,E**) controls. (**G–L**) Gain of Ecad function expands the prohemocyte population. *dome-Gal4* females were crossed to (**G,I,K**) control (+) or (**H,J,L**) *UAS-Ecad* males. (**G–L**) Over-expression of the Ecad wild-type transgene increased the number of cells expressing (**H**) Ecad, (**J**) Odd and (**L**) Ptc compared to (**G,I,K**) controls. Yellow dotted lines delineate the entire lymph gland; white dotted lines delineate the prohemocyte pool. Scale bars: 50 µm. (**M**) Histogram showing the percentage of labeled cells per primary lymph gland lobe; Ecad (n = 12), Odd (n = 15), and Ptc (n = 12) in control (+) and Ecad knockdown (Ecad^RNAi^) lymph glands. (**N**) Histogram showing the percentage of labeled cells per primary lymph gland lobe; (Ecad; n = 12), (Odd; n = 16), and (Ptc; n = 12) in control (+) and Ecad over-expression genetic backgrounds. Two tailed Student’s t-test; error bars show standard deviation; P values are as shown.

E-cadherin is expressed in medullary zone prohemocytes and downregulated in differentiating cortical zone hemocytes [4], [34]. However, it is not known if E-cadherin actually regulates prohemocyte multipotency and differentiation. To begin to address this question, we investigated if E-cadherin is required to maintain the prohemocyte pool. RNAi technology was combined with the UAS/Gal4 binary system to knockdown E-cadherin in medullary zone prohemocytes. Specifically, we used the *domeless-Gal4* (*dome-Gal4*) driver to express a *UAS-E-cadherin^RNAi^* transgene in prohemocytes (Figure 1). We first established that E-cadherin expression was significantly reduced compared to *dome-Gal4* heterozygous controls (Figure 1A,B,M). Next, we tested if E-cadherin maintains the prohemocyte pool by assaying marker expression in lymph glands with targeted knockdown of E-cadherin. Odd is a marker that is used to identify prohemocytes [41]. Targeted knockdown of E-cadherin significantly reduced the Odd expression domain compared to controls (Figure 1C,D,M). We verified this result by conducting two additional experiments. First, we used another prohemocyte-specific driver, *Tep4-Gal4*, to drive *E-cadherin^RNAi^* transgene expression. Second, we used the *dome-Gal4* driver to express an *E-cadherin^RNAi^* transgene from another source (Bloomington Stock Center). In both cases, we observed a significant decrease in the Odd expression domain (Figure S1). To confirm that the decrease in marker expression was representative of prohemocyte loss and not just loss of Odd expression, we assayed for expression of the prohemocyte-specific marker, Patched (Ptc) [5]. The Ptc expression domain was also significantly reduced with knockdown of E-cadherin in prohemocytes compared to controls (Figure 1E,F,M). Collectively, these results indicate that E-cadherin maintains the prohemocyte pool. In addition, these results predict that over-expression of E-cadherin would increase prohemocyte number. To test this hypothesis, we used the UAS/Gal4 system with the *dome-Gal4* driver to over-express E-cadherin in prohemocytes. We first showed that over-expression of E-cadherin significantly increased the number of E-cadherin-positive cells (Figure 1G,H,N). Next, we showed that over-expression of E-cadherin produced a significant increase in both the Odd and Ptc expression domains (Figure 1I–L,N). Thus, E-cadherin maintains the prohemocyte pool while over-expression expands the prohemocyte pool.

**Figure 2:**
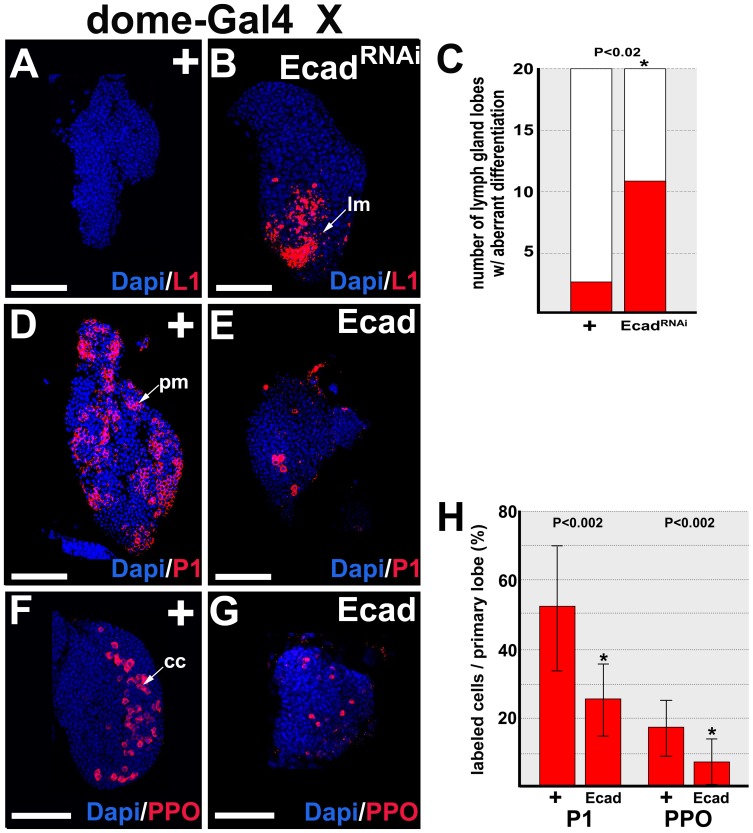
E-cadherin limits prohemocyte differentiation. (**A–C**) Loss of E-cadherin (Ecad) function results in aberrant lamellocyte differentiation. *dome-Gal4* females were crossed to control (+) or *UAS-Ecad^RNAi^* (Ecad^RNAi^) males to knockdown Ecad in prohemocytes. (**A**) Lamellocytes (lm) are rarely observed in controls. (**B**) *dome-Gal4* driven knockdown of Ecad (Ecad^RNAi^) results in a dramatic increase in the number of lamellocytes. Lamellocytes were identified using the cell-specific marker, L1. Scale bars: 50 µm. (**C**) Histogram showing the number of primary lymph gland lobes with aberrant lamellocyte differentiation was significantly greater when Ecad expression was knocked down. Two-tailed Fisher’s exact test; P value is as shown; n = 20. (**D–H**) Gain of Ecad function limits prohemocyte differentiation. *dome-Gal4* females were crossed to (**D,F**) control (+) or (**E,G**) *UAS-Ecad* (Ecad) males. Over-expression of Ecad limited (**E**) plasmatocyte (pm) and (**G**) crystal cell (cc) differentiation compared to (**D,F**) controls. Plasmatocytes were identified using the cell-specfic marker, P1. Crystal cells were identified using the cell-specific marker Prophenoloxydase (PPO). Scale bars: 50 µm. (**H**) Histogram showing the percentage of plasmatocytes (P1; n = 17) or crystal cells (PPO; n = 17) was significantly greater in control (+) than in Ecad over-expression genetic backgrounds. Two-tailed Student’s t-test; error bars show standard deviation; P values are as shown.

In differentiating cortical zone hemocytes, E-cadherin is downregulated [4], [16], [34], [41]. This suggests that E-cadherin blocks differentiation to preserve the prohemocyte pool. To test this hypothesis, we evaluated blood cell differentiation in lymph glands with prohemocyte-specific knockdown of E-cadherin. Lamellocytes are rarely observed under steady-state conditions [9]; however, targeted knockdown of E-cadherin produced a significant increase in lamellocyte differentiation (Figure 2A,B,C). Again, we verified this result using two additional combinations of prohemocyte-specific Gal4 drivers and *UAS-E-cadherin^RNAi^* transgenes from the VDRC and Bloomington stock centers. In both cases, we observed a significant increase in lamellocyte differentiation compared to controls (Figure S2A). However, knockdown of E-cadherin in prohemocytes did not produce a significant increase in the number of crystal cells or plasmatocytes (Figure S2B–F). Nevertheless, E-cadherin blocks prohemocyte differentiation in *dome-Gal4* driven over-expression of *UAS-E-cadherin* (Figure 1I–L,N). This predicts that over-expression of E-cadherin should block production of both crystal cells and plasmatocytes. Indeed, we observed that both cell types were significantly reduced under these conditions (Figure 2D–H). Thus, over-expression of E-cadherin expands the prohemocyte pool at the expense of crystal cells and plasmatocytes. These results indicate that E-cadherin is required to maintain prohemocyte multipotency by blocking entrance into the differentiation pathway.

### Mis-expression of E-cadherin in Differentiating Cortical Zone Hemocytes Upregulates Patched Expression and Disrupts Zonal Boundaries

With the loss of E-cadherin, we observed aberrant lamellocyte differentiation and a severe reduction in prohemocyte number. Furthermore, over-expression of E-cadherin increased the size of the prohemocyte pool. Together, these findings suggest that E-cadherin expression has a profound effect on the prohemocyte transcriptional landscape. Based on these observations, we tested if mis-expressing E-cadherin in differentiating cortical zone hemocytes would be sufficient to drive these cells towards a multipotent state.

**Figure 3:**
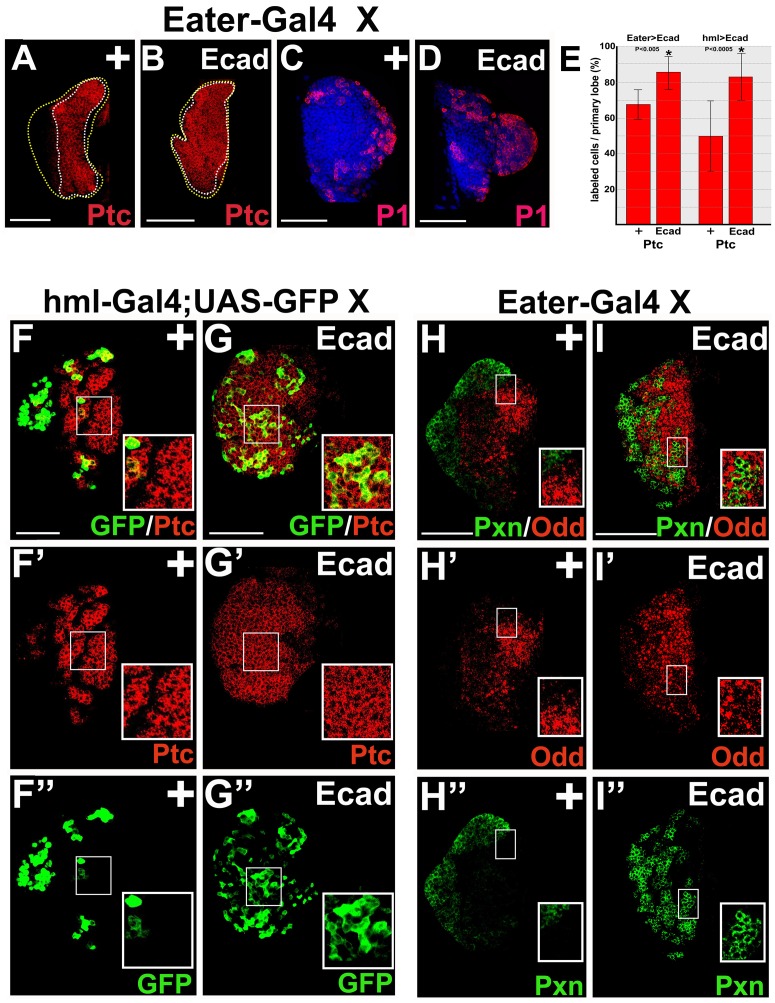
E-cadherin mis-expression in the cortical zone upregulates Patched expression and perturbs zonal boundaries. (**A–E**) Mis-expression of E-cadherin (Ecad) in cortical zone hemocytes expands the Ptc expression domain, but has no effect on plasmatocyte differentiation. *Eater-Gal4* females were crossed to (**A,C**) control (+) or (**B,D**) *UAS-Ecad* males. (**B**) *Eater-Gal4* driven Ecad significantly increased the Ptc expression domain compared to (**A**) the control. (**C,D**) However, *Eater-Gal4* driven Ecad had no effect on plasmatocyte differentiation. Plasmatocytes were identified using the cell-specific marker, P1. Yellow dotted lines delineate the entire lymph gland; white dotted lines delineate the Ptc expression domain. Scale bars: 50 µm. (**E**) Histogram showing the percentage of Ptc labeled cells per primary lymph gland lobe in *Eater-Gal4* or *hml-Gal4* driven Ecad lymph glands compared to controls (+). Two tailed Student’s t-test; error bars show standard deviation; P values are as shown; n = 12. (**F–G”**) *hml-Gal4* driven Ecad expression produces an increase in the following: 1) the Ptc expression domain; 2) the number of cells that are positive for both Ptc and *hml-Gal4* driven GFP (Ptc^+^; hml>GFP^+^); and 3) the number of hml>GFP^+^ hemocytes within the medial region of the lymph gland. (**F,G**) Lymph glands co-stained for Ptc and GFP. (**F’,F”,G’,G”**) The same lymph glands showing only (**F’,G’**) Ptc staining and (**F”,G”**) GFP staining. (**F–G”**) Insets are an enlarged region of medullary zone showing co-expression of Ptc and GFP. (**H–I”**) *Eater-Gal4* driven Ecad expression has no effect on Odd or Pxn expression, but produces an increase in the number of Pxn^+^ hemocytes within the medial region of the lymph gland. (**H,I**) Lymph glands co-stained for Odd and Pxn. (**H’,H”,I’,I”**) The same lymph glands showing only (**H’,I’**) Odd staining and (**H”,I”**) Pxn staining. (**H–I”**) Insets are an enlarged region showing expression of Pxn^+^ hemocytes or Odd^+^ prohemocytes. Scale bars: 50 µm.

We first established that E-cadherin could be upregulated in the lymph gland cortical zone by using *Eater-Gal4* [49] to drive *UAS-E-cadherin* (Figure S3A–C). The level of E-cadherin mis-expression was much greater than the level of endogenous E-cadherin (Figure S3A,B). Therefore, in order to view both mis-expression and endogenous expression, the micrograph was overexposed (Figure S3C). We then assessed the effect of mis-expressing E-cadherin on medullary zone prohemocytes. In addition to *Eater-Gal4*, we also used *hemolection-Gal4* (*hml-Gal4*) [16] to mis-express *UAS-E-cadherin* in cortical zone hemocytes (Figures 3 and S3). Prohemocytes were monitored using Ptc and Odd. Both *Eater-Gal4* and *hml-Gal4* driven *UAS-E-cadherin* mis-expression resulted in a statistically significant increase in the Ptc expression domain compared to controls (Figure 3A,B,E,F,G). We also observed an increase in the number of cells that expressed both Ptc and the differentiation marker, *hml-Gal4* driving *UAS-GFP* (hml>GFP; Figure 3F–G”). In contrast, we did not observe a significant increase in Odd-expressing prohemocytes under these conditions (Figure 3H,I and Figure S3D,E,H). These results indicate that mis-expression of E-cadherin can upregulate a prohemocyte-specific marker in differentiating cortical zone cells but not produce an overall expansion of the prohemocyte pool.

Next, we assessed the effect of mis-expressing E-cadherin on the cortical zone hemocyte population. Hemocytes were monitored using P1 (Nimrod), Peroxidasin (Pxn), and hml>GFP [5], [16], [41], [44], [47]. We did not observe a significant difference in the expression of any hemocyte markers when either *hml-Gal4* or *Eater-Gal4* was used to drive *E-cadherin* expression in the cortical zone (Figure 3C,D,F–I and Figure S3F–H). These results show that mis-expression of E-cadherin in the cortical zone does not block differentiation, and they differ from those that show over-expression of E-cadherin in medullary zone cells expanded the prohemocyte pool and blocked differentiation (Figures 1I,J,N and 2D–H). These functional differences indicate that E-cadherin must act in conjunction with other prohemocyte factors to expand the prohemocyte pool and block differentiation.

We also observed differentiating cortical zone cells within the presumptive medullary zone of lymph glands. When *Eater-Gal4* was used to mis-express E-cadherin in the cortical zone, we observed differentiating Pxn-positive cell clusters intermingled with medullary zone Odd-positive prohemocytes in the medial region of lymph glands (Figure 3I–I”). Additionally, cells that co-expressed the medullary zone marker Ptc and the cortical zone marker hml>GFP were observed throughout the lymph gland, most notably in the medullary zone (Figure G–G”). These data are in stark contrast to control lymph glands (hml-Gal4/+ or Eater-Gal4/+; Figure 3F,H) and previously published results [4], [16] that show Pxn-positive and hml>GFP-positive cells remained primarily within the cortical zone. Thus, upregulation of E-cadherin in cortical zone cells appears to disrupt zonal boundaries. Interestingly, loss of E-cadherin can also disrupt zonal boundaries [16]. Collectively, these observations indicate that precise expression of E-cadherin is a key factor in maintaining differentiating cells within the cortical zone and undifferentiated prohemocytes within the medullary zone.

### E-cadherin Acts Downstream of U-shaped to Block Lamellocyte Differentiation

**Figure 4:**
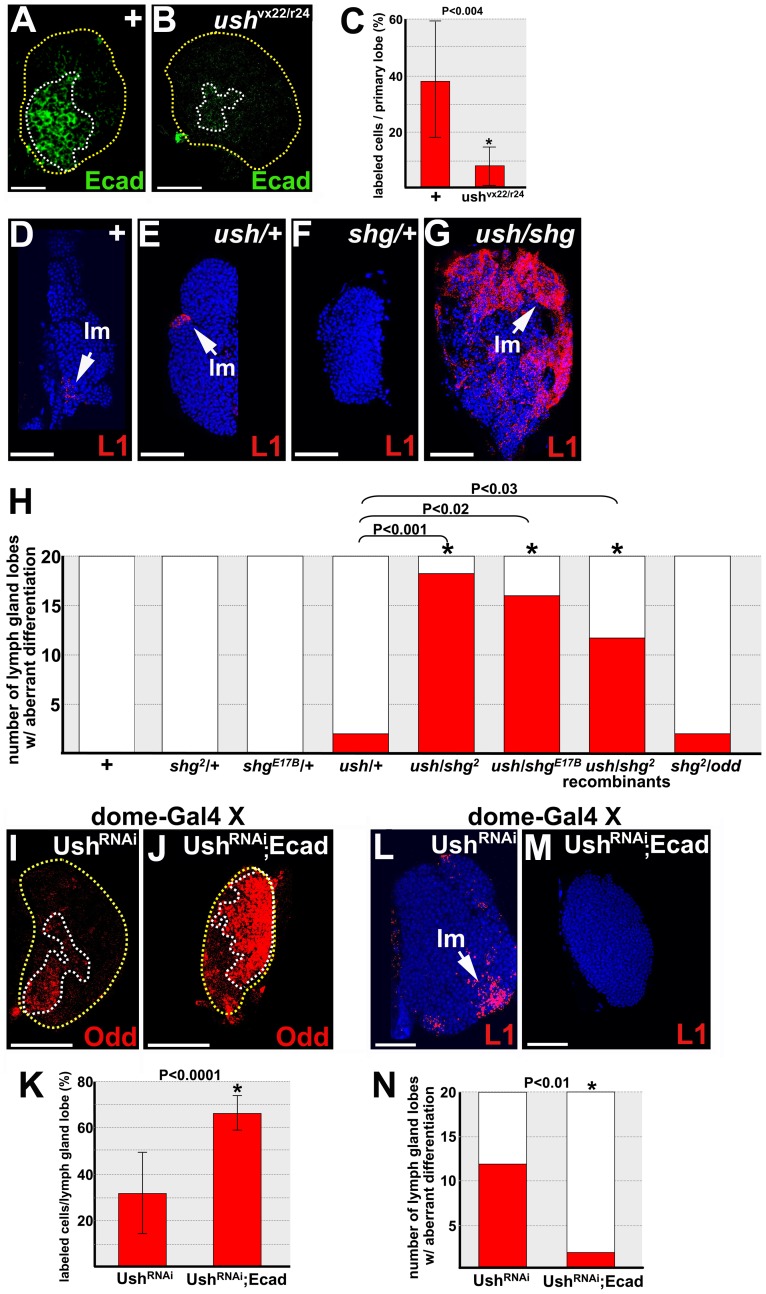
E-cadherin is a downstream effector of U-shaped. (**A,B**) U-shaped (Ush) is required for E-cadherin (Ecad) expression in early-third instar larvae. (**B**) Ecad expression is reduced in *ush^vx22/r24^* trans-heterozygotes compared to (**A**) controls (+). Yellow dotted lines delineate the entire lymph gland; white dotted lines delineate the Ecad expression domain. (**C**) Histogram showing the percentage of Ecad-expressing cells per primary lobe was significantly reduced in *ush* ^vx22/r24^ lymph glands compared to controls (+). Two-tailed Student’s t-test; error bars show standard deviation; P value is as shown; n = 16. (**D–G**) Ush and Ecad function in the same pathway. (**D–F**) Loss of one copy of either *ush* or *shg* does not produce increased lamellocyte (lm) differentiation. (**G**) In contrast, *ush/shg* transheterozygotes show a dramatic increase in lamellocyte differentiation. (**H**) Histogram showing the number of primary lymph gland lobes exhibiting lamellocyte differentiation in wild-type (+), *yw;shg^2^*/+, *yw;shg^E17B^*/+, *yw;ush^vx22^*/+, *yw;ush^vx22^/shg^2^*, *yw;ush^vx22^/shg^E17B^*, *yw;ush^vx22^/shg^2^* recombinants and *yw;odd^01863^*/*shg^2^* larvae. Two-tailed Fisher’s exact test; P values are as shown; n = 20. (**I–N**) Ecad rescues loss of Ush function. *dome-Gal4* females were crossed to *UAS-Ush^RNAi^* (Ush^RNAi^) or *UAS-Ush^RNAi^*;*UAS-Ecad^RNAi^* (Ush^RNAi^;Ecad) males. Odd expression was reduced in (**I**) lymph glands that expressed Ush^RNAi^ alone compared to (**J**) lymph glands that expressed both Ush^RNAi^ and Ecad. Yellow dotted lines delineate the entire lymph gland; white dotted lines delineate the Odd expression domain. (**K**) Histogram showing the percentage of Odd labeled cells was significantly reduced in Ush^RNAi^ lymph glands compared to Ush^RNAi^;Ecad lymph glands. Two tailed Student’s t-test; error bars show standard deviation; P values are as shown; n = 14. (**L**) Lamellocyte (lm) differentiation increased in lymph glands that expressed Ush^RNAi^ compared to (**M**) lymph glands that expressed both Ush^RNAi^ and Ecad. (**N**) Histogram showing lamellocyte differentiation significantly increased in Ush^RNAi^ lymph glands compared to Ush^RNAi^;Ecad lymph glands. Two-tailed Fisher’s exact test; P value is as shown; n = 20. Scale bars: A,B 10 µm; D–G,I,J,L,M 50 µm.

Previously, we showed that Ush promotes prohemocyte multipotency and blocks differentiation [34]. Two observations suggested that E-cadherin acts downstream of Ush to control prohemocyte fate choice. First, both Ush and E-cadherin loss-of-function phenotypes exhibit reduced prohemocyte numbers and increased lamellocyte differentiation (Figures 1C–M and 2A–C) [34], [50]. Second, loss of Ush function leads to loss of E-cadherin expression [34]. If E-cadherin acts downstream of Ush to block lamellocyte differentiation, then loss of Ush function should result in loss of E-cadherin expression early in lymph gland development prior to the onset of lamellocyte differentiation. Ush is first detected in the lymph gland during the late-second or early-third larval instar [34], [50]. Thus, we assayed E-cadherin expression in lymph glands from early-third instar *ush^vx22/r24^* hypomorphs shortly after the onset of Ush expression. We observed that E-cadherin expression was significantly reduced and that this occurred prior to the onset of lamellocyte differentiation (Figure 4A–C). Differentiation was monitored using the lamellocyte-specific marker, L1 (data not shown). Given that Ush is required to maintain the prohemocyte pool, loss of E-cadherin expression could have resulted from a reduction in the number of prohemocytes. To test this possibility, we assayed for Odd expression in lymph glands from early-third instar *ush^vx22/r24^* hypomorphs. We observed that Odd expression levels were not significantly different from age-matched controls (Figure S4). However, it should be noted that Odd expression is significantly downregulated in late-third instar *ush^vx22/r24^* hypomorphs due to reduction of the prohemocyte pool [41]. Collectively, these observations indicate that Ush maintains E-cadherin expression in prohemocytes. In contrast, loss of E-cadherin function did not result in loss of Ush expression. We used the UAS/Gal4 system with the *Tep4-Gal4* driver and *UAS-E-cadherin^RNAi^* to knockdown E-cadherin expression in prohemocytes and did not observe a significant difference in Ush expression (Figure S5). These results suggest that Ush acts upstream of E-cadherin and E-cadherin is a downstream target of Ush.

We determined that Ush and E-cadherin act in the same regulatory pathway by assaying for a genetic interaction between *ush^vx22^* and *shg^2^* with aberrant blood cell differentiation being the end-point. The results of these analyses showed that *shg*/*ush* trans-heterozygotes exhibited aberrant lamellocyte differentiation, whereas larvae that were singularly heterozygous for either *ush* or *shg* exhibited lamellocyte numbers that were similar to wild-type controls (Figure 4D–H). We confirmed these results using an alternate allele of *shg* (*shg^E17B^*; Figure 4H). Additionally, we tested *ush^vx22^* and *shg^2^* alleles that were recombined with wild-type chromosomes and again showed that trans-heterozygotes produced aberrant lamellocyte differentiation, whereas heterozygotes did not (Figure 4H). This indicated that the increase in lamellocyte production was due to an interaction between *ush* and *shg*, rather than interactions between other mutant genes that may reside on each of their respective chromosomes. We hypothesize that aberrant lamellocyte differentiation occurs in trans-heterozygotes because the effective dose of E-cadherin is reduced by lowering the dose of the upstream regulator, Ush.

Ush and E-cadherin do not appear to function with Odd, despite the fact that all three factors are required to block lamellocyte differentiation. Previously, we showed that *ush*/*odd* trans-heterozygotes do not produce increased lamellocyte differentiation [41]. Likewise, we showed that *shg*/*odd* trans-heterozygotes do not produce increased lamellocyte differentiation (Figure 4H). This is consistent with the notion that Ush and E-cadherin act in the same pathway, whereas neither function with Odd to block lamellocyte differentiation.

Finally, if Ush maintains E-cadherin expression in prohemocytes to block differentiation, then ectopic expression of E-cadherin should rescue loss of Ush function. We tested this hypothesis using the UAS/Gal4 binary system and the *dome-Gal4* driver to co-express *UAS-E-cadherin* and *UAS-Ush^RNAi^* transgenes in prohemocytes. We assayed for both Odd expression and lamellocyte production in late stage lymph glands. Odd expression was significantly increased while lamellocyte differentiation was significantly reduced when *UAS-E-cadherin* and *UAS-Ush^RNAi^* were co-expressed compared to expression of *UAS-Ush^RNAi^* alone (Figure 4I–N). Collectively, these expression and functional analyses indicate that E-cadherin acts downstream of Ush to maintain multipotency by blocking differentiation.

**Figure 5:**
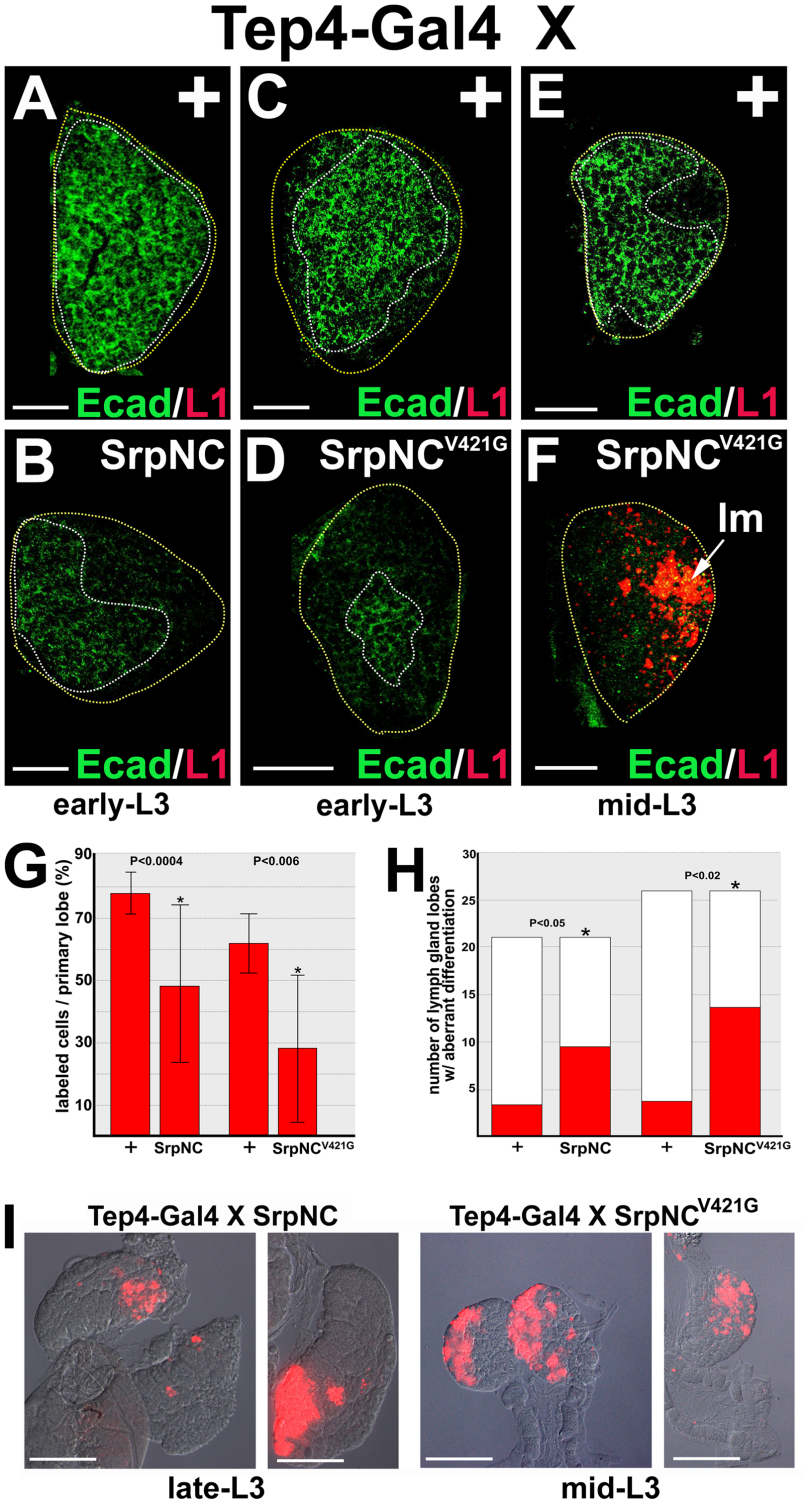
Serpent represses E-cadherin and promotes lamellocyte differentiation. Forced expression of SrpNC or the Ush non-binding mutant form of SrpNC (SrpNC^V421G^) represses E-cadherin (Ecad) expression and promotes lamellocyte differentiation. (**A–D**) Forced expression of SrpNC or SrpNC^V421G^ represses Ecad expression differentiation in lymph glands from early-third instar (L3) larvae. (**E,F**) Forced expression of SrpNC^V421G^ promotes lamellocyte differentiation during mid-L3. *Tep4-Gal4* females were crossed to (**A,C,E**) control (+), (**B**) *UAS-srpNC* (SrpNC) or (**D,F**) *UAS-srpNC^V421G^* (SrpNC^V421G^) males. Ecad expression and lamellocyte (lm) production were assessed using immunofluorescent staining. Lamellocytes were identified with the specific marker, L1. Yellow dotted lines delineate the entire lymph gland; white dotted lines delineate the prohemocyte pool. Scale bars: A,B 10 µm; C–F 20 µm. (**G**) Histogram showing the percentage of Ecad labeled cells per primary lymph gland lobe in control (+), SrpNC (n = 16), and SrpNC^V421G^ (n = 15) lymph glands. Two-tailed Student’s t-test; error bars show standard deviation; P values are as shown. (**H**) Histogram showing the number of primary lymph gland lobes exhibiting aberrant lamellocyte differentiation in control (+), SrpNC (n = 21), and SrpNC^V421G^ (n = 26) lymph glands. Two-tailed Fisher’s exact test; P values are as shown. (**I**) Lamellocyte differentation is not observed until late-L3 in *Tep4-Gal4* driven *UAS-srpNC* (SrpNC). In *Tep4-Gal4* driven *UAS-srpNC^V421G^* (SrpNC^V421G^), lamellocyte differentiation is observed in mid-L3, which is earlier than *Tep4-Gal4* driven *UAS-srpNC* (SrpNC). Lamellocytes are identified with the specific marker, L1. Scale bars: 50 µm.

Ush functions in a variety of tissues by interacting with a specific GATA binding partner [40], [48], [51]–[55]. During hematopoiesis, Ush has been shown to interact with the canonical two zinc-finger isoform of Srp (SrpNC) to regulate Srp-activated transcription [40], [48], [50]–[55]. Moreover, Ush and Srp are co-expressed in the medullary zone [34], [48], [52], [56] and may form a GATA:FOG complex that promotes E-cadherin expression. Srp functions at the apex of hematopoiesis to maintain and expand the prohemocyte pool [52], [55], [57]–[60], and is also required later in hematopoiesis to drive the differentiation of specific cell types [40], [52], [54], [55], [61]. As a result, loss of Srp function profoundly disrupts normal hematopoiesis. In order to determine whether Ush and Srp form a complex that maintains E-cadherin expression, we tested the effect of expressing either SrpNC or a Ush non-binding mutant version of SrpNC on E-cadherin expression and lamellocyte production (Figure 5). FOG factors interact with their GATA partners by binding the N-terminal zinc finger. Seven of the 25 amino acids within the zinc finger are conserved and required for GATA:FOG complex binding, including SrpNC valine 421 [62]–[64]. In this mutant version of SrpNC, the conserved valine 421 was changed to glycine (SrpNC^V421G^) [40]. We observed that *Tep-Gal4* driven prohemocyte-specific ectopic-expression of either wild-type *UAS-SrpNC* (Tep4>SrpNC) or Tep4>SrpNC^V421G^ resulted in a significant decrease in E-cadherin expression early in lymph gland development (Figure 5A–D,G). Additionally, ectopic expression of either the wild-type or mutant version of SrpNC produced aberrant lamellocyte differentiation later in lymph gland development (Figure 5E,F,H,I). However, Tep4>SrpNC^V421G^ reduced E-cadherin expression and increased lamellocyte differentiation to a greater degree than did Tep4>SrpNC. Tep4>SrpNC^V421G^ reduced E-cadherin on average by 53% compared to only 37% by Tep4>SrpNC (Figure 5G). Lamellocyte differentiation was observed on average 20 hours earlier in Tep4>SrpNC^V421G^ lymph glands compared to Tep4>SrpNC lymph glands (Figure 5I). In Tep4>SrpNC^V421G^ lymph glands, robust lamellocyte differentiation was observed as early as the mid-third larval instar (92 to 100 hours after egg laying). This is in contrast to Tep4>SrpNC lymph glands where lamellocytes were not observed until the late-third larval instar (112 to 120 hours after egg laying; Figure 5I). Moreover, lamellocyte differentiation appeared to be greater in Tep4>SrpNC^V421G^ lymph glands (Figure 5I). Thus, SrpNC was less effective than SrpNC^V421G^ in repressing E-cadherin expression and promoting lamellocyte differentiation. This is consistent with the notion that Ush binding inhibits the activity of SrpNC. Collectively, these results suggest that when Srp is not bound to Ush, it represses E-cadherin expression. In contrast, when Ush binds SrpNC, it inhibits the ability of SrpNC to repress E-cadherin expression and, thereby, limits lamellocyte differentiation.

## Discussion

In this report, we show that E-cadherin is required to maintain *Drosophila* prohemocyte multipotency and block differentiation. Additionally, mis-expression of E-cadherin in cortical zone hemocytes upregulates the medullary zone marker Ptc and disrupts the boundary between the lymph gland cortical and medullary zones. Our studies also indicate that the *Drosophila* GATA transcriptional co-factor Ush is a positive regulator of E-cadherin expression. As such, E-cadherin is a downstream target of Ush in the maintenance of prohemocyte multipotency. One the other hand, the *Drosophila* GATA factor SrpNC represses E-cadherin expression to promote lamellocyte differentiation. Thus, Ush most likely maintains E-cadherin expression by blocking the inhibitory activity of SrpNC.

RNAi knockdown of E-cadherin increased lamellocyte differentiation and reduced prohemocyte numbers. However, it had no apparent effect on terminally differentiated plasmatocytes or crystal cell numbers, despite the fact that over-expression of E-cadherin blocked the differentiation of plasmatocytes and crystal cells. These data suggest that knockdown of E-cadherin changes the transcriptional landscape such that prohemocytes enter the lamellocyte differentiation pathway, while having no apparent effect on plasmatocyte and crystal cell differentiation. This could be explained by the fact that the plasmatocyte and crystal cell lineages reached the committed precursor stage before reduction in E-cadherin expression altered the prohemocyte transcriptional landscape. In addition, lamellocytes can be produced at the expense of plasmatocytes and crystal cells [34], [41], [50], [65], [66]. Thus, it is also possible that increased numbers of prohemocytes were poised to enter the plasmatocyte and crystal cell differentiation pathway, but were re-directed to the lamellocyte pathway in response to E-cadherin loss. In any case, loss of E-cadherin may alter the prohemocyte transcriptional landscape in such a way to activate lamellocyte-specific stress response signals.

### E-cadherin Maintains the Potency of Mammalian ESCs and *Drosophila* Prohemocytes

We observed that the function of E-cadherin in prohemocytes was similar to that reported for mammalian pluripotent stem cells. In both systems, E-cadherin blocks differentiation and maintains the progenitor pool. Studies using gene expression profiling indicate that E-cadherin regulates the transcriptional networks associated with maintenance of mammalian ESC pluripotency [25]. In particular, E-cadherin may regulate extrinsic signals that converge to maintain the expression of core pluripotency factors, including Oct, Nanog and Sox2 [25]. Likewise, our work suggests that E-cadherin plays a central role in maintaining the prohemocyte multipotency gene regulatory program and inhibiting the differentiation program. This function of E-cadherin is supported by the following observations: First, loss of E-cadherin expression in medullary zone prohemocytes produces a dramatic shift in developmental potential, resulting in a significant increase in the number of rare immune-responsive lamellocytes. Second, over-expression of E-cadherin in medullary zone prohemocytes expands this population of undifferentiated cells. In both cases, changes in the level of E-cadherin alter the gene regulatory landscape to produce profound changes in cell fate choice. Overall, this indicates that a causal relationship exists between E-cadherin-mediated cell adhesion and the gene regulatory networks that control the choice between progenitor potency and differentiation.

### Commonalities between Mis-expression of E-cadherin in *Drosophila* Hemocytes and Mammalian Cellular Reprogramming

E-cadherin acts as part of a network that includes the pluripotency factors Sox, Klf4 and Myc. These factors produce fully reprogrammed iPSCs [21], [23]. In *Drosophila*, E-cadherin is most likely required to act with other factors to promote prohemocyte multipotency. This hypothesis is supported by our new findings that show over-expression of E-cadherin in prohemocytes blocks hemocyte differentiation and expands the progenitor pool. In contrast, we showed that mis-expression of E-cadherin in differentiating hemocytes neither increased the prohemocyte pool nor blocked differentiation. These different outcomes may be due to the availability of required prohemocyte co-factors that work with E-cadherin to promote multipotency.

E-cadherin is upregulated as part of an initial mesenchymal to epithelial transition that is required for mammalian cellular reprogramming [21], [23], [28], [67]–[69]. Upregulation of E-cadherin coincides with an increase in pluripotency markers [67], [68] and, as a result, an increase in the number of intermediate cell types. These cell types include reprogramming-competent cells that emerge as a subset of the heterogeneous population [67], [68], [70], [71]. Likewise, the mis-expression of E-cadherin in *Drosophila* hemocytes produced an increase in the number of intermediate cell types that expressed both the differentiation marker hml>GPF and the multipotency marker Ptc. Furthermore, these cells lost their zonal identity. These changes in the genotype and phenotype of differentiating hemocytes may be an initial step towards reprogramming competency. Thus, in both systems, upregulation of E-cadherin may orchestrate key changes that prime differentiating cells for cellular reprogramming.

These similarities in E-cadherin function suggest that components of the E-cadherin-based regulatory network are conserved across taxa. The *Drosophila* model system is ideally suited to identify the molecular details of this network. The advantage of using the fly system is that the interactions between E-cadherin and the extracellular microenvironment are maintained. As a result, the information gained from studies using *Drosophila* genetics can complement mammalian tissue culture-based approaches that currently rely on a limited representation of the extracellular environment.

### E-cadherin Expression Levels may be Regulated by GATA:FOG Complex Formation

Studies from our laboratory identified Ush as a key factor in the maintenance of prohemocyte multipotency [34]. We have extended this finding by using genetic interaction studies and epistatic analyses to show that Ush is required for E-cadherin expression and that E-cadherin is a downstream effector of Ush.

In addition, our work indicates that Ush promotes E-cadherin expression by blocking the inhibitory activity of SrpNC. We showed that E-cadherin is repressed by over-expressing SrpNC in medullary zone prohemocytes, which is followed by aberrant lamellocyte differentiation. Importantly, ectopic expression of the Ush non-binding mutant form of SrpNC (SrpNC^V421G^) also reduced E-cadherin expression and increased lamellocyte differentiation but to a greater degree than did over-expression of wild-type SrpNC. This would be expected if Ush blocks the activity of SrpNC. Finally, ectopic expression of either the wild-type or mutant version of SrpNC mimics the Ush loss of function phenotype. These data indicate that increasing the level of unbound SrpNC reduces E-cadherin expression. Furthermore, Ush maintains E-cadherin expression by binding SrpNC and inhibiting its ability to repress E-cadherin expression. Consequently, this model predicts that the control of GATA:FOG complex formation regulates E-cadherin expression levels and, thereby, the choice between maintenance of prohemocyte potency and differentiation.

### GATA Factors have Become Recognized as Important Regulators of E-cadherin Function Across Taxa

Downregulation of E-cadherin is a defining characteristic of epithelial-mesenchymal transition (EMT), which is a critical process in normal development and drives metastatic cancer [72], [73]. Thus, identifying regulators of E-cadherin is a key to understanding the molecular mechanisms that govern these processes. While factors such as Twist, Snail and Zeb are widely recognized as negative regulators of E-cadherin, recent studies have shown that GATA factors can both positively and negatively control E-cadherin function as a means of regulating EMT [36], [38], [39]. Two studies have shown that two different GATA factors, GATA-3 and tricho-rhino-phalangeal syndrome type 1 (TRPS1), activate E-cadherin gene expression in breast cancer cells. This leads to a reversal of EMT and an inhibition of metastasis [38], [39]. Another study showed that the GATA factor, Srp, promotes EMT in the developing *Drosophila* gut by repressing E-cadherin function [36]. Srp has no effect on the level of E-cadherin expression, but rather represses *crumbs* expression. This ultimately leads to the loss of adherins junction formation and relocation of E-cadherin from the membrane to the cytoplasm. This regulatory mechanism is evolutionarily conserved because GATA-4 and -6 block expression of the mammalian *crumbs* homolog in MDCK cells, which promotes the relocation of E-cadherin [36]. In contrast, our studies suggest that Srp represses E-cadherin expression in *Drosophila* prohemocytes and, importantly, Ush appears to reverse this process. We showed that increasing the level of unbound SrpNC or decreasing the level of Ush results in a reduction in the number of cells that express E-cadherin. Moreover, we did not observe translocation of E-cadherin from the membrane to the cytoplasm under these circumstances. Whether Srp-driven repression of E-cadherin in prohemocytes constitutes an EMT-like event is not known. However, two observations suggest that this might be the case. First, loss of Ush function in prohemocytes results in increased matrix metalloproteinase (MMP) expression (Gao and Fossett, unpublished), which is a key characteristic of EMT. Second, the loss of E-cadherin leads to differentiation in both prohemocytes and mammalian ESCs. Differentiation of ESCs has been characterized as an EMT-like event [72], [73]. Thus, this common function may be indicative of a conserved mechanism. Nevertheless, understanding how GATA and FOG regulate this important adhesion molecule will help identify gene networks that control normal development and metastatic cancer.

In summary, we have shown that E-cadherin maintains prohemocyte potency and blocks differentiation. Moreover, our studies suggest that E-cadherin functions in the *Drosophila* hematopoietic system in a manner that resembles its role in mammalian pluripotent stem cells. Importantly, we have extended our previous work by showing that Ush maintains prohemocyte multipotency and blocks differentiation by acting through E-cadherin. This may involve blocking the inhibitory activity of SrpNC. As such, we have provided evidence that control of GATA:FOG complex formation regulates E-cadherin expression and, thereby, the choice between multipotency and differentiation in *Drosophila* prohemocytes.

## Supporting Information

Figure S1 **E-cadherin knockdown reduces the Odd-skipped-expression domain.** Odd expression was assessed in lymph glands with alternate combinations of prohemocyte Gal4 drivers and *UAS-Ecadherin^RNAi^* (Ecad^RNAi^) transgenes. Histogram showing the percentage of Odd labeled cells per primary lymph gland lobe with the following two combinations of prohemocyte-specific Gal4 drivers and *UAS-Ecad^RNAi^* targets: 1) *dome-Gal4* driven *UAS-Ecad^RNAi^* from the Bloomington Stock Center (Dome>Ecad^RNAi^ Bloomington; n = 12); 2) *Tep4-Gal4* driven *UAS-Ecad^RNAi^* from VDRC (Tep4>Ecad^RNAi^ VDRC; n = 17). Two tailed Student’s t-test; error bars show standard deviation; P values are as shown.
(TIF)

Figure S2 **E-cadherin knockdown alters blood cell differentiation in lymph glands.** (**A**) Knockdown of E-cadherin significantly increased lamellocyte differentiation. Lamellocyte differentiation was assessed in lymph glands with alternate combinations of prohemocyte Gal4 drivers and *UAS-Ecadherin^RNAi^* (Ecad^RNAi^) transgenes. Histogram showing the number of primary lymph gland lobes exhibiting aberrant lamellocyte differentiation with the following two combinations of prohemocyte-specific Gal4 drivers and *UAS-Ecad^RNAi^* (Ecad^RNAi^) targets: 1) *Tep4-Gal4* driven *UAS-Ecad^RNAi^* from VDRC (Tep4>Ecad^RNAi^ VDRC); 2) *dome-Gal4* driven *UAS-Ecad^RNAi^* from the Bloomington Stock Center (dome>Ecad^RNAi^ Bloomington). Two-tailed Fisher’s exact test; P values are as shown; n = 20. (**B–F**) Knockdown of E-cadherin has no effect on plasmatocyte or crystal cell differentiation. *Tep4-Gal4* females were crossed to (**B,D**) control (+) or (**C,E**) *UAS-E-cadherin^RNAi^* (Ecad^RNAi^) males. (**B,C**) Plasmatocytes were identified using the cell-specific marker, P1 and (**D,E**) crystal cells were identified using the cell-specific marker Prophenoloxydase (PPO). Yellow dotted lines delineate the lymph gland. Scale bars: 50 µm. (**F**) Histogram showing that the percentage of plasmatocytes (P1; n = 19) or crystal cells (PPO; n = 20) was not significantly different when E-cadherin was knocked down (Ecad^RNAi^) and control (+) lymph glands. Two-tailed Student’s t-test; error bars show standard deviation; P values are as shown.
(TIF)

Figure S3 **Mis-expression of E-cadherin does not increase the numbers of Odd-, Pxn- or P1-expressing blood cells.** (**A–C**) Eater-Gal4 driven mis-expression of E-cadherin (Ecad) upregulates E-cadherin expression in the cortical zone. *Eater-Gal4* females were crossed to (**A**) control (+) or (**B,C**) *UAS-Ecad* males. (**C**) Over-exposed micrograph showing endogenous Ecad expression. (**D–G**) Odd and Pxn expression in lymph glands from animals with *hml-Gal4* driven *UAS-Ecad*. *hml-Gal4* females were crossed to (**D,F**) control (+) or (**E,G**) *UAS-Ecad* males. *hml-Gal4* driven mis-expression of E-cadherin did not increase the number of (**E**) Odd- or (**G**) Pxn-expressing cells compared to controls (**D,F**). However, there was an increase in the number of Pxn-expressing cells in the medullary zone of lymph glands from animals with (**G**) mis-expressed Ecad compared to (**F**) controls (marked with arrow). Yellow dotted lines delineate the entire lymph gland; white dotted lines delineate the prohemocyte pool. Scale bars: 25 µm. (**H**) Histogram showing the percentage of labeled cells per primary lymph gland lobe in *Eater-Gal4* or *hml-Gal4* driven Ecad lymph glands compared to controls (+). Mis-expression of Ecad did not produce a significant increase in the number of Odd-, Pxn-, or P1-expressing cells. Two tailed Student’s t-test; error bars show standard deviation; P values are as shown. *Eater-Gal4* driven mis-expression of Ecad (Odd, n = 12; Pxn, n = 12; P1, n = 17). *hml-Gal4* driven mis-expression of Ecad (Odd, n = 17; Pxn n = 12).
(TIF)

Figure S4 **Ush is not required for Odd-skipped expression in early-third instar larvae.** (**A,B**) Odd-skipped (Odd) expression was assessed in control and *ush^vx22/r24^* trans-heterozygous lymph glands from early-third instar larvae. (**A**) Odd expression was not reduced in *ush^vx22/r24^* trans-heterozygotes compared to (**B**) controls. Yellow dotted lines delineate the entire lymph gland; white dotted lines delineate the prohemocyte pool. Scale bars: 10 µm. (**C**) Histogram showing the percentage of Odd-expressing cells per primary lobe was not significantly different in *ush* ^vx22/r24^ lymph glands compared to controls (+). Two-tailed Student’s t-test; error bars show standard deviation; P value is as shown; n = 22.
(TIF)

Figure S5 **E-cadherin knockdown does not affect Ush expression.** *Tep4-Gal4* females were crossed to (**A**) control (+) or (**B**) *UAS-E-cadherin^RNAi^* (Ecad^RNAi^) males. Scale bars: 50 µm. (**C**) Histogram showing that the level of Ush expression was not significantly different when E-cadherin was knocked down (Ecad^RNAi^) and control (+) lymph glands. Two-tailed Student’s t-test; error bars show standard deviation; P values are as shown; n = 19.
(TIF)
