# Supplementary figures and images for: Drosophila E-Cadherin Functions in Hematopoietic Progenitors to Maintain Multipotency and Block Differentiation
Source: PLoS One. 2013 Sep 5;8(9):e74684. doi: 10.1371/journal.pone.0074684 (PMC3764055; doi:10.1371/journal.pone.0074684)

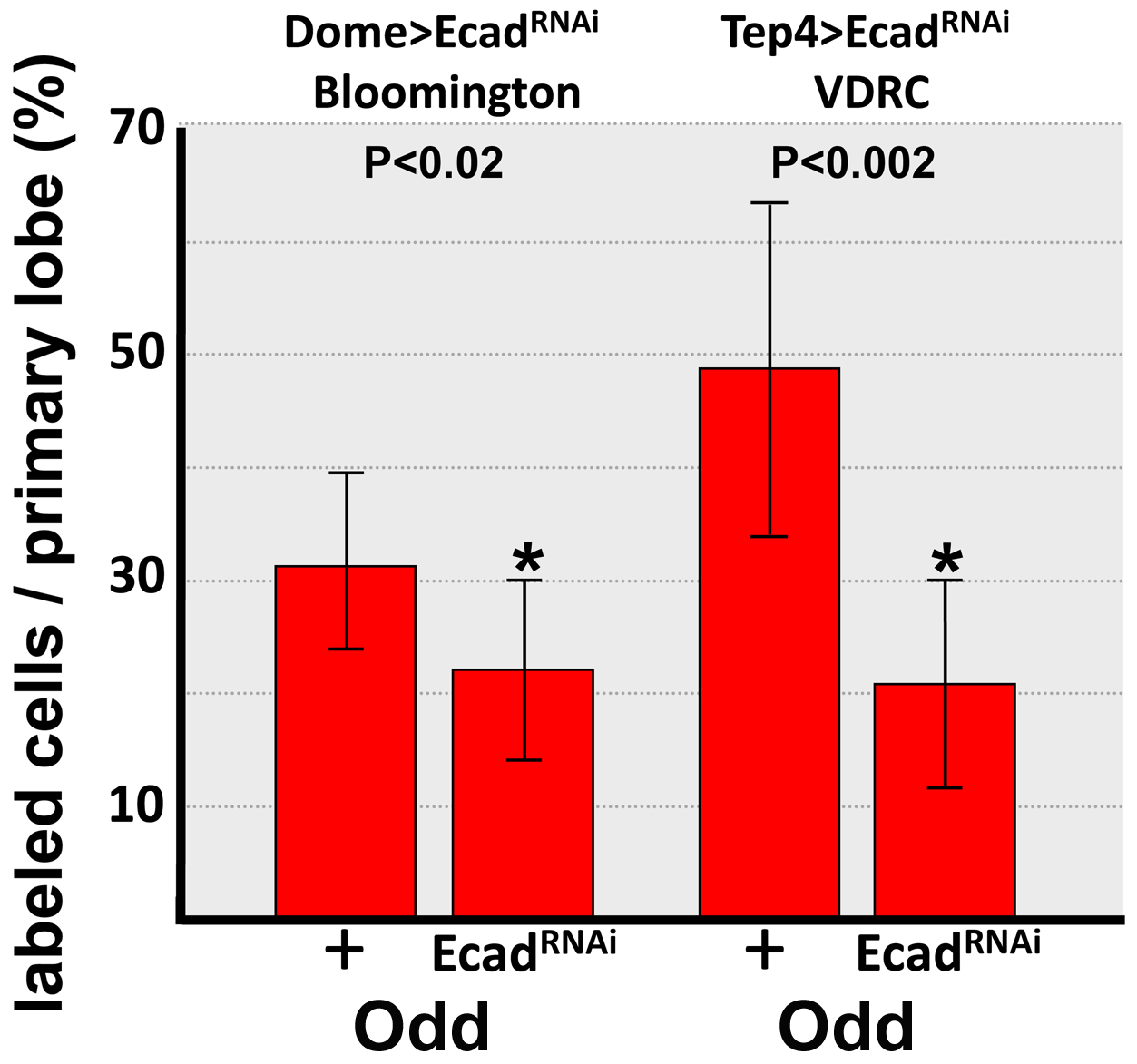

Supplement: Figure S1 — E-cadherin knockdown reduces the Odd-skipped-expression domain. Odd expression was assessed in lymph glands with alternate combinations of prohemocyte Gal4 drivers and UAS-EcadherinRNAi (EcadRNAi) transgenes. Histogram showing the percentage of Odd labeled cells per primary lymph gland lobe with the following two combinations of prohemocyte-specific Gal4 drivers and UAS-EcadRNAi targets: 1) dome-Gal4 driven UAS-EcadRNAi from the Bloomington Stock Center (Dome>EcadRNAi Bloomington; n = 12); 2) Tep4-Gal4 driven UAS-EcadRNAi from VDRC (Tep4>EcadRNAi VDRC; n = 17). Two tailed Student’s t-test; error bars show standard deviation; P values are as shown. (TIF) [file pone.0074684.s001.tif]

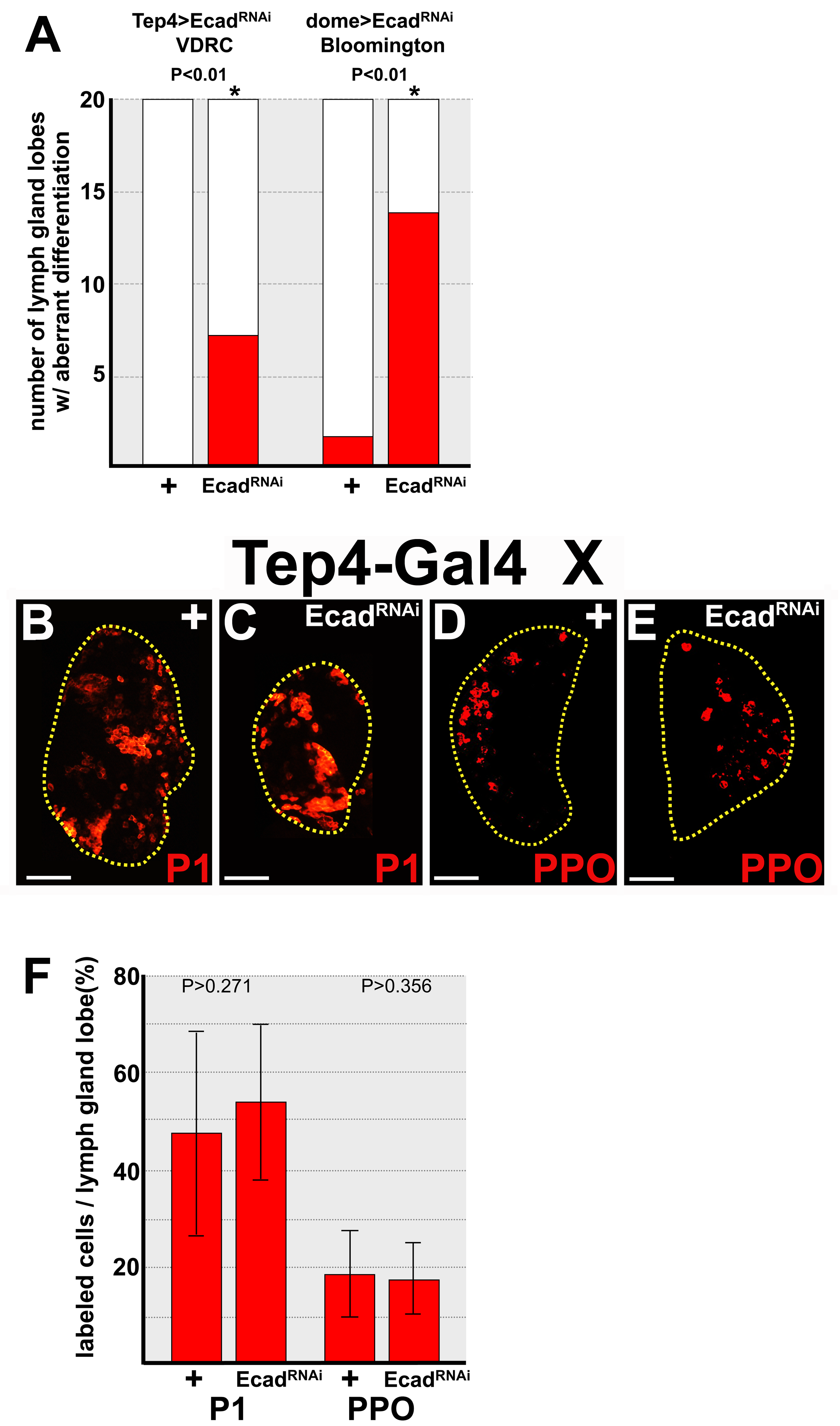

Supplement: Figure S2 — E-cadherin knockdown alters blood cell differentiation in lymph glands. (A) Knockdown of E-cadherin significantly increased lamellocyte differentiation. Lamellocyte differentiation was assessed in lymph glands with alternate combinations of prohemocyte Gal4 drivers and UAS-EcadherinRNAi (EcadRNAi) transgenes. Histogram showing the number of primary lymph gland lobes exhibiting aberrant lamellocyte differentiation with the following two combinations of prohemocyte-specific Gal4 drivers and UAS-EcadRNAi (EcadRNAi) targets: 1) Tep4-Gal4 driven UAS-EcadRNAi from VDRC (Tep4>EcadRNAi VDRC); 2) dome-Gal4 driven UAS-EcadRNAi from the Bloomington Stock Center (dome>EcadRNAi Bloomington). Two-tailed Fisher’s exact test; P values are as shown; n = 20. (B–F) Knockdown of E-cadherin has no effect on plasmatocyte or crystal cell differentiation. Tep4-Gal4 females were crossed to (B,D) control (+) or (C,E) UAS-E-cadherinRNAi (EcadRNAi) males. (B,C) Plasmatocytes were identified using the cell-specific marker, P1 and (D,E) crystal cells were identified using the cell-specific marker Prophenoloxydase (PPO). Yellow dotted lines delineate the lymph gland. Scale bars: 50 µm. (F) Histogram showing that the percentage of plasmatocytes (P1; n = 19) or crystal cells (PPO; n = 20) was not significantly different when E-cadherin was knocked down (EcadRNAi) and control (+) lymph glands. Two-tailed Student’s t-test; error bars show standard deviation; P values are as shown. (TIF) [file pone.0074684.s002.tif]

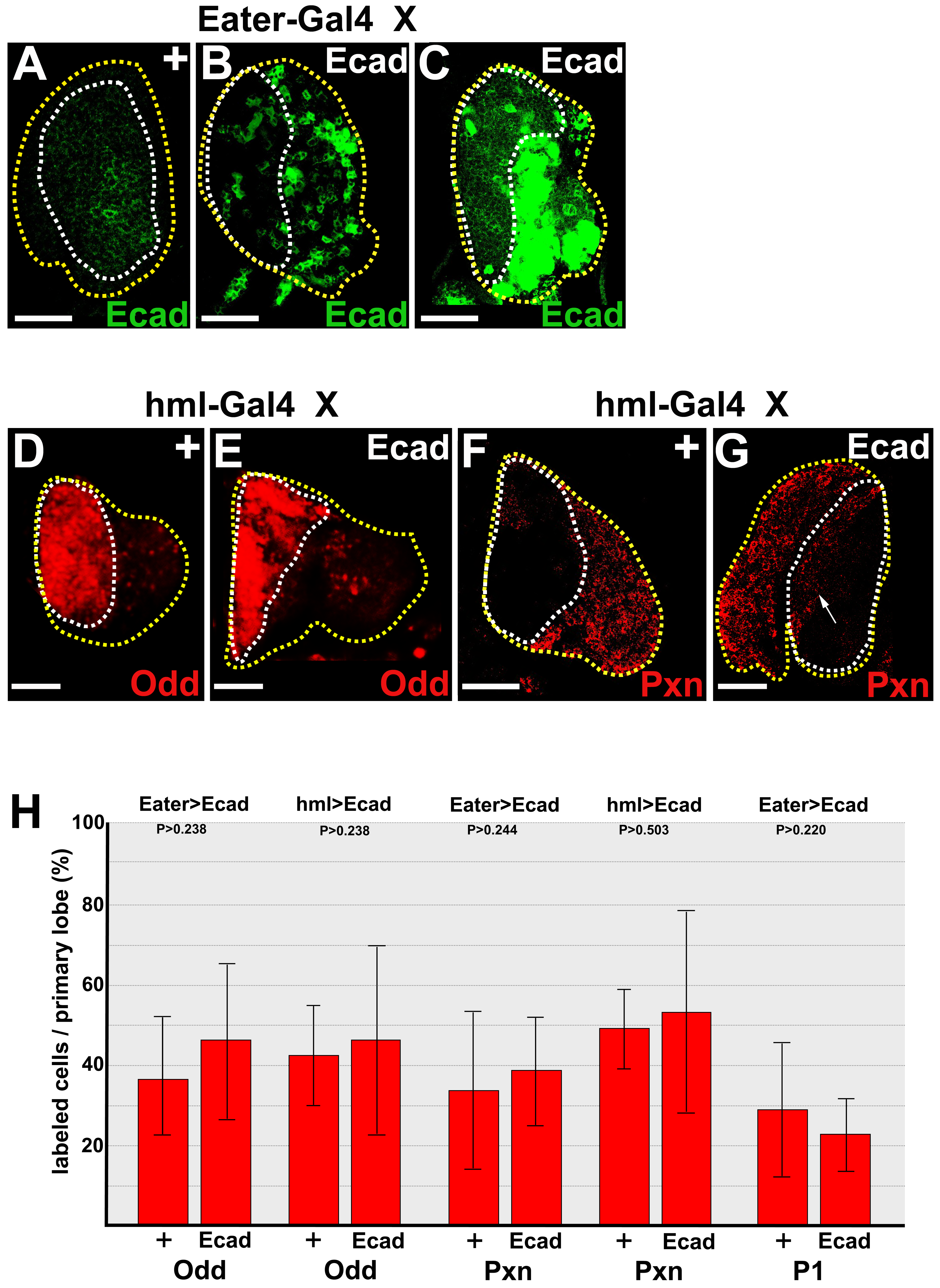

Supplement: Figure S3 — Mis-expression of E-cadherin does not increase the numbers of Odd-, Pxn- or P1-expressing blood cells. (A–C) Eater-Gal4 driven mis-expression of E-cadherin (Ecad) upregulates E-cadherin expression in the cortical zone. Eater-Gal4 females were crossed to (A) control (+) or (B,C) UAS-Ecad males. (C) Over-exposed micrograph showing endogenous Ecad expression. (D–G) Odd and Pxn expression in lymph glands from animals with hml-Gal4 driven UAS-Ecad. hml-Gal4 females were crossed to (D,F) control (+) or (E,G) UAS-Ecad males. hml-Gal4 driven mis-expression of E-cadherin did not increase the number of (E) Odd- or (G) Pxn-expressing cells compared to controls (D,F). However, there was an increase in the number of Pxn-expressing cells in the medullary zone of lymph glands from animals with (G) mis-expressed Ecad compared to (F) controls (marked with arrow). Yellow dotted lines delineate the entire lymph gland; white dotted lines delineate the prohemocyte pool. Scale bars: 25 µm. (H) Histogram showing the percentage of labeled cells per primary lymph gland lobe in Eater-Gal4 or hml-Gal4 driven Ecad lymph glands compared to controls (+). Mis-expression of Ecad did not produce a significant increase in the number of Odd-, Pxn-, or P1-expressing cells. Two tailed Student’s t-test; error bars show standard deviation; P values are as shown. Eater-Gal4 driven mis-expression of Ecad (Odd, n = 12; Pxn, n = 12; P1, n = 17). hml-Gal4 driven mis-expression of Ecad (Odd, n = 17; Pxn n = 12). (TIF) [file pone.0074684.s003.tif]

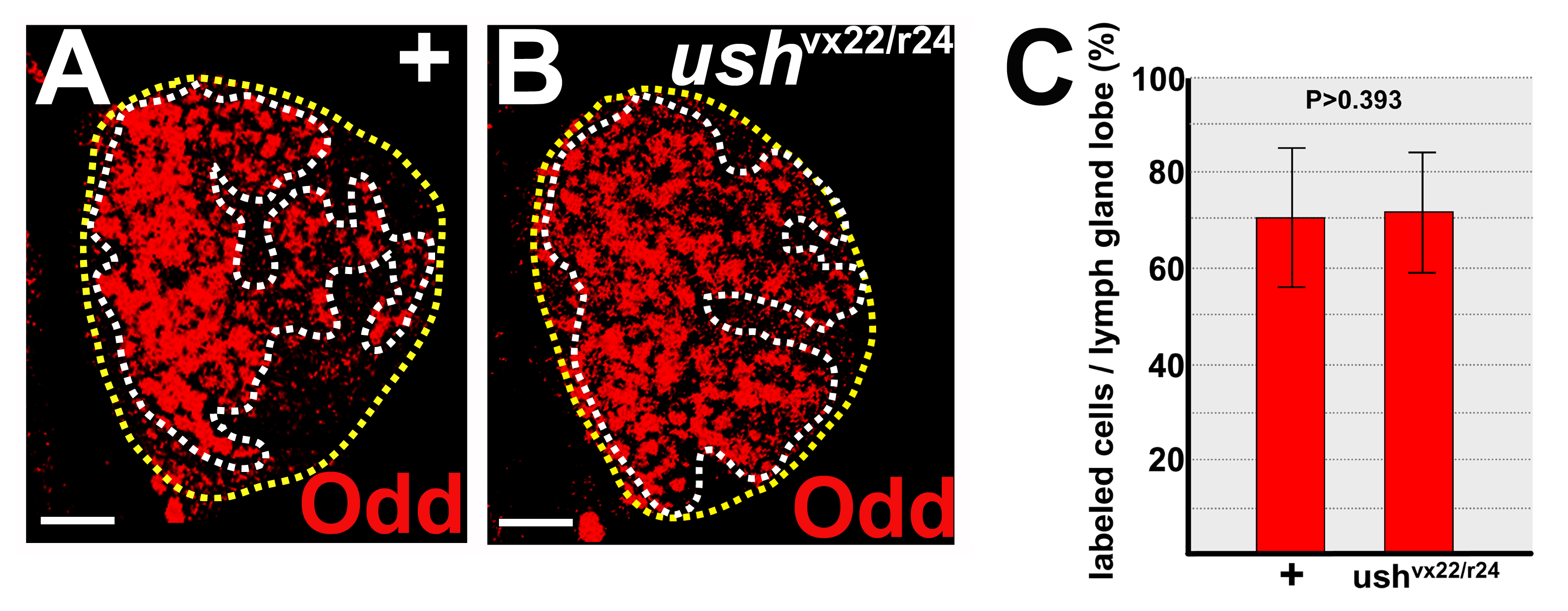

Supplement: Figure S4 — Ush is not required for Odd-skipped expression in early-third instar larvae. (A,B) Odd-skipped (Odd) expression was assessed in control and ushvx22/r24 trans-heterozygous lymph glands from early-third instar larvae. (A) Odd expression was not reduced in ushvx22/r24 trans-heterozygotes compared to (B) controls. Yellow dotted lines delineate the entire lymph gland; white dotted lines delineate the prohemocyte pool. Scale bars: 10 µm. (C) Histogram showing the percentage of Odd-expressing cells per primary lobe was not significantly different in ush vx22/r24 lymph glands compared to controls (+). Two-tailed Student’s t-test; error bars show standard deviation; P value is as shown; n = 22. (TIF) [file pone.0074684.s004.tif]

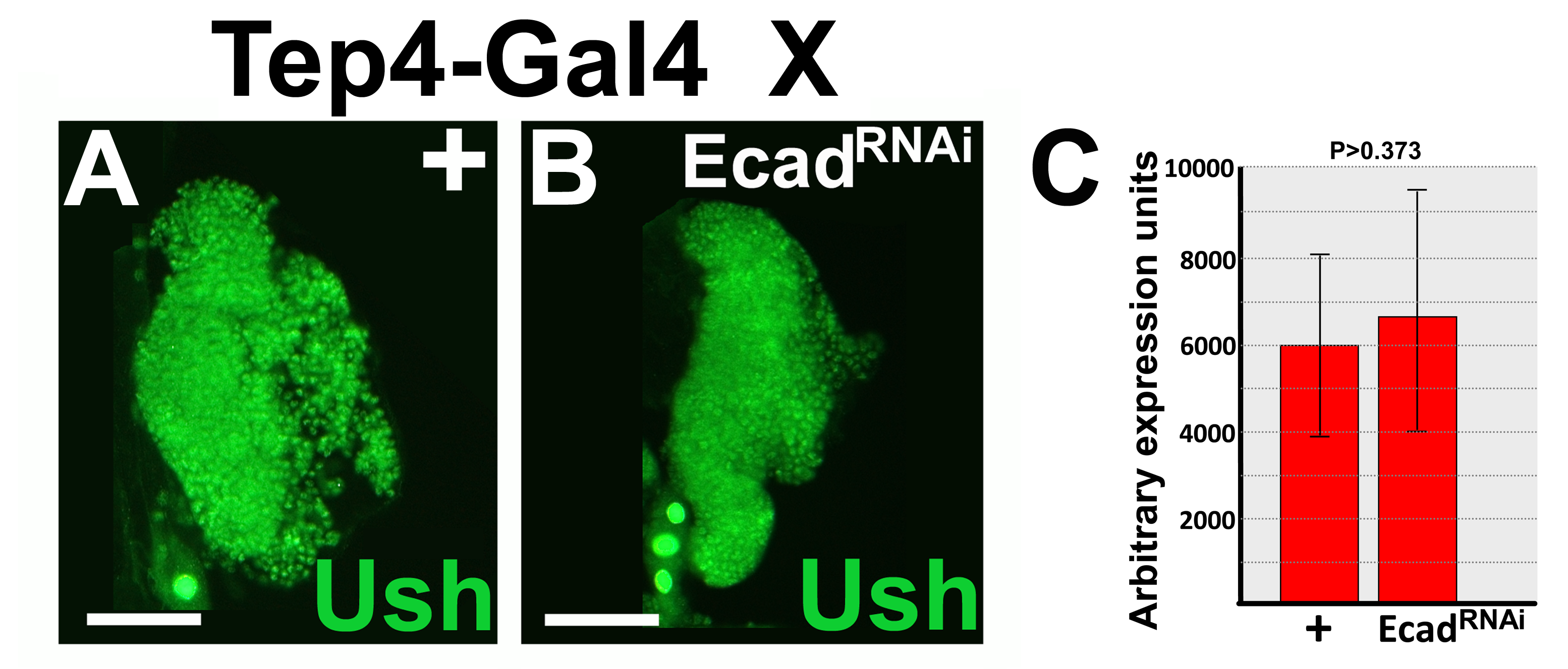

Supplement: Figure S5 — E-cadherin knockdown does not affect Ush expression. Tep4-Gal4 females were crossed to (A) control (+) or (B) UAS-E-cadherinRNAi (EcadRNAi) males. Scale bars: 50 µm. (C) Histogram showing that the level of Ush expression was not significantly different when E-cadherin was knocked down (EcadRNAi) and control (+) lymph glands. Two-tailed Student’s t-test; error bars show standard deviation; P values are as shown; n = 19. (TIF) [file pone.0074684.s005.tif]
